# Food Safety Practices and Associated Factors in Food Operators: A Cross-Sectional Survey in the Students' Cafeteria of Woldia University, North Eastern Ethiopia

**DOI:** 10.1155/2022/7400089

**Published:** 2022-12-09

**Authors:** Silamlak Birhanu Abegaz

**Affiliations:** Woldia University, Faculty of Natural and Computational Sciences, Department of Biology, Ethiopia

## Abstract

The food safety issue is often overlooked in countries where food shortages, natural disasters, political tensions, and other major concerns dominate government and media agendas. As a result, the current study sought to assess food safety practices and associated factors among food handlers at Woldia University's student cafeteria. An institutional cross-sectional study was conducted between May and July 2021, and a sample of 291 subjects was recruited through a simple random sampling technique. Questionnaires, focus group discussions, interviews, and observation methods were used as data collection tools. Data analysis was performed using Statistical Package for the Social Sciences (SPSS) version 20 software. Bivariate and multivariate logistic regressions were used to determine the effect of various factors on the outcome variable and control for confounding effects. *p* < 0.05 was considered statistically significant. However, some variables are significant even at *p* < 0.001. The results were described by frequencies and percentages. The current study found that the factors of not having had food hygiene training (AOR = 2.111, 95% CI = (1.029 − 4.428)), less than or equal to one year of work experience (AOR = 3.070, 95% CI = (2.020 − 10.246)), poor knowledge (AOR = 1.285, 95% CI = (0.125 − 0.849)) and poor attitude (AOR = 1.190, 95% CI = (1.361 − 9.393)), not keeping cooked food at a safe temperature (AOR = 3.037, 95% CI = (1.021 − 12.096)), failure to respect the safety of cooking utensils and surfaces (AOR = 2.022, 95% CI = (1.551 − 9.689)), insufficient cleanliness of eating areas (AOR = 2.430, 95% CI = (1.983 − 6.217)), not covering hair when cooking food (AOR = 5.903, 95% CI = (2.243 − 9.621)), and not washing hands before starting to handle food (AOR = 10.019, 95% CI = (4.031 − 24.063)) were statistically associated with unhygienic food handling practices. The results of this study indicated that the state of food safety practices was poor. Therefore, food safety assurance must comply with modern food safety frameworks such as Hazard Analysis and Critical Control Points (HACCP). In addition, addressing knowledge and skill gaps among food handlers, regular inspection services, and effective enforcement of food safety regulations are extremely essential. Finally, future studies should focus on enumerating bacteria and protozoa in unsanitary foods and utensils.

## 1. Introduction

In a province where food shortages, infectious diseases, political uncertainty, natural disasters, and other major concerns dominate government and media agendas, the importance of food safety often goes unspoken [1]. However, food safety is vital for developing countries like Ethiopia due to the aggravating impact of the above concerns [1]. Foodborne infections are common on emerging continents, so widespread disease and premature death were common [2]. There is an increased incidence of diarrheal infections in African children of 3.3-4.1 cases per child per year. It is expected that 800,000 children in Africa die each year from diarrhea and dehydration [2]. People with malaria, tuberculosis, HIV/AIDS, and other diseases in developing countries are more vulnerable to being weakened by unsafe foods because their immune systems are more likely to deteriorate [3]. Therefore, ensuring food safety is essential to improve the quality of life of the victims [3]. Similarly, people with foodborne illnesses are more likely to contract other infectious diseases. The primary objective of the Food Safety Regulations is to reduce the risk of illness from unprotected food. Unfortunately, food protection measures in most developing countries produce the expected results. Therefore, food safety compliance requires good alignment between regulatory design and desired outcomes [4]. Food safety measures are known to be ineffective in ensuring food safety in the various food establishments in Ethiopia.

The food safety control system in Ethiopia is largely traditional, inadequate, and lacks clear provisions for modern food safety frameworks such as HACCP. Furthermore, the food laws conflicted with international standards (Codex). Frankly, food laws create inevitable misunderstandings between food processors, inspectors, producers, and suppliers [5]. Comprehensible national policies for food safety are the building blocks of holistic food hygiene management [6]. However, the government of Ethiopia has paid no attention to the impact of food safety on public health. Hence, it is given low priority in national decision-making. For example, street foods that are fresh, processed, and directly consumed food products remain the least controlled or fall outside the scope of the official control system, either by food hygiene authorities or environmental health experts. While risk assessments are necessary for the formulation of relevant food safety legislation as well as food inspection priorities and other food safety policies [7], almost all African countries have faced various challenges in terms of complications for food safety and inspection issues [8].

Consequently, the level of contamination and the toxicological risks of different foods have been affected every year. In addition, the food control mechanism in Africa in general has been confronted with fraudulent political practices. Even the professional rank of inspectors does not reflect their responsibility. The lack of inspection equipment, transport, and assessment procedures were other constraints to the impossibility of carrying out the inspections. Although few food inspections have been carried out in the capitals and major cities of Ethiopia, no inspection system has been established in the small communities and rural areas of the country [5]. Food can be adulterated in three ways: (i) poor personal hygiene, (ii) time or temperature control, and (iii) cross-contamination [9]. Therefore, poor food safety practices can be a major reason for lowering food quality. Several reports indicate that poor food safety practices by food handlers are responsible for 10-20% of food-related illnesses [10].

Another study conducted in Latin America confirmed that improper food safety practices account for 97% of foodborne illnesses in grocery stores and cafes [11]. Furthermore, substantial evidence from primary sources confirmed that food processors regularly made mistakes in food safety.

This shows that grocers should have the greatest share in ensuring food hygiene during the food handling process [12]. According to the previous study by Negassa et al. [5] in Addis Ababa, only 27.4% of grocers have implemented adequate food safety practices in different food establishments. Consequently, food safety practices in Ethiopia are even worse. Studies by Al-Kandari et al. [13] have shown that parameters such as knowledge, attitudes, and practices related to the sanitary status of food establishments in all developing countries need further investigation. Some studies suggest that a lack of awareness among grocers leads to poor hygiene practices [14]. On the other hand, some studies available in emerging markets have shown that grocers rarely implemented good handling practices despite insufficient food safety awareness [15]. Several factors, including personal hygiene, and organizational and socioeconomic outlook, are said to impact the ability of food processors to handle food safely [16]. Foodborne infections and deaths in Africa are on the rise due to poor handling practices in catering and canteens [14]. Due to unsanitary food handling practices, approximately two billion infections are associated with foodborne illness [17]. Particularly in mass catering, food processors are the main stakeholders who contaminate food as a biological or physical vector of many disease-causing microbes [17].

Foods can be contaminated during cooking, packaging, storage, or loading [18]. Therefore, major disease outbreaks are due to improper food handling practices. The Codex Nutrition Committee has confirmed that inappropriate food handling is the primary reason for foodborne infections and that hand sanitation is a significant risk factor in preventing food adulteration [19]. Improper food handling was probably associated with 97% of all foodborne illnesses [19]. According to the European Food Safety Authority [20], approximately 48.7% of foodborne infections are related to food supply facilities such as catering and canteens. Explicit reasons for foodborne infections were improper handling, health and hygiene of personnel, and the presence of microbes such as *Escherichia coli* and *Staphylococcus aureus* on various parts of the body of food handlers [21].

Food poisoning also occurs when eating or drinking food adulterated with microbes or their toxins. Food contamination can result from inadequate protective measures, unsanitary personalities, and cross-contamination between food processing surfaces and utensils, or personnel can protect microorganisms [22]. Food contamination leads to a deterioration in food quality and thus leads to quantity concerns, economic concerns, and community health concerns [23].

Many reports have shown that approximately 420,000 deaths can occur each year due to foodborne infections [22]. Several scholars have shown that food and beverage outlets in different cities or towns of Ethiopia were in a higher standard of unsanitary conditions. Evidence reported from the city of Motta [24] and Addis Ababa [25] showed the underrated unsanitary locations of catering areas and equipment.

Collectively, the physical environments of cafeterias and kitchen restaurants in these urban centers appeared to be unsanitary, with inadequate sanitation, poor hand-washing services, and improper disposal of liquid and solid waste [26]. Although prodigious efforts have been made in Ethiopia to alleviate complications related to food safety, including higher institutions, food safety and personal hygiene practices of grocers remain a challenge and cause for great concern [26]. In Ethiopia, many people are thought to suffer and even die from ingesting contaminated food and water every year. Additionally, college catering establishments have been linked to various foodborne outbreaks. Therefore, higher education students in Ethiopia are at-risk groups who might be susceptible to various foodborne diseases; because they share common food and drink facilities and serve crockery or cutlery, cross-contamination and infection rates between groups or individuals may be higher just by considering them. However, the recent study conducted by Alemnew et al. [27] revealed the presence of intestinal parasitic infections among food handlers in the study area; gaps in food safety practices and associated factors at food processors have not been filled. Therefore, this study might be interesting to reflect the current information on the hygienic conditions of food handlers and food and beverage outlets in the Woldia University student canteen. Therefore, the present study is aimed at evaluating the level of food safety practices and associated factors among food handlers working in the Woldia University student cafeteria.

## 2. Material and Methods

### 2.1. Description of the Study Area

Woldia University is one of the dynamic growth oriented higher education institutions in Ethiopia. It was established in the city of Woldia, the capital of North Wollo Zone, Amhara Regional State. It is about 521 km from Addis Ababa, the capital of Ethiopia. The university has two campuses, i.e., the main campus is called Woldia University and the other is Mersa Agriculture Campus. It is 30 km from the main campus. However, the present study was conducted on the main campus. At present, the university employed over 9023 students and 595 food processing workers serving in the student cafeteria.

### 2.2. Study Design and Period

An institutional cross-sectional survey was conducted between May and July 2021.

### 2.3. Source Population

All food workers working in the student canteen of Woldia University were considered as the source population.

### 2.4. Inclusion and Exclusion Criteria

Food operators who have direct contact with food, restaurants, and eating places were in demand. In addition, those who were randomly selected constitute a study population. Food safety operators working on the Mersa campus, those who are disabled and seriously ill as well as absentee workers were excluded from the study.

### 2.5. Sample Size Determination

The sample size was determined using a single population ratio formula that accounted for Cochran's hypotheses [28]: good food safety practices of food handlers 14% (*p* = 0.14); *Z* = 95% confidence interval equal to 1.96; *d* is an acceptable margin of error (*d* = 0.05). So, the equation would be

(1) $$\begin{array}{c} n=\frac{{\left(Z\alpha /2\right)}^{2}\times \text{P}\left(1-\text{P}\right)}{d^{2}}\text{ thus},n=\frac{{\left(1.96\right)}^{2}\times 0.14\left(1-0.14\right)}{{\left(0.05\right)}^{2}}=185. \end{array}$$

The adjustment was made by adding the 5% nonresponse and multiplying by 1.5 the design effect gives the final sample size of the study, which are 291.

### 2.6. Sampling Technique

For good sampling technique, total numbers and document listings of food processors (595) were obtained from the Woldia University Office of Student Affairs and Facilities. Study participants were then selected from lists using a simple random sampling technique following the lottery method. Document lists (name archives) of food processors were used as a sampling framework.

### 2.7. Data Collection Tools

The questionnaires were developed by Abdi et al. [26] and Abate et al. [29] adapted and used for data collection. In addition, development was also done by reviewing previously published studies (Supplementary Material (available here)). Questions from the English version were converted to the local language (Amharic) and then back to English by language experts to check for consistency and clarity of the question and used for this study. The questionnaire covered sociodemographic characteristics, food safety practices of grocers, and the physical health status of food establishments. Respondents who meet the inclusion criteria can participate in the questionnaire survey.

#### 2.7.1. Focus Group Discussion

This was conducted with a total of fifty food workers grouped into four focus group discussions with 8-13 people each. The participants were of different religions, ages, genders, classes, and educational levels in the social composite. Issues such as food safety culture and effective sanitation measures were discussed in each focus group session. The FGD was guided by the mediator who guided the conversation using a checklist prepared by the Principal Investigator for this particular study. Additionally, interview processes were conducted with Food Safety Managers, Student Affairs, and Facility Office Managers to supplement the information gathered during the discussion. Finally, observation was performed using an observation checklist to minimize the overestimation of actual activities and self-desirability bias among respondents.

### 2.8. Data Administration and Quality Control

Successful training was provided to two biology undergraduates who were proficient in field data collection, a food and environmental health professional, and a superintendent engaged in data collection. Explanations were provided for each specific question included in the data collection tool. The validity of the tool was ensured through pilot tests. In case of inconsistency, corrections were made before the actual data collection. The principal investigator reviewed all study modules to confirm the completeness and consistency of the information.

### 2.9. Study Variables

Food safety practices (good or bad) were a dependent variable, while respondents' sociodemographic characteristics and other factors related to food safety practices were considered independent variables.

### 2.10. Data Analysis

Data were first checked for completeness, then coded, entered, and analyzed using SPSS (Statistical Package for the Social Sciences) version 20 software. Binary logistic regression was used to determine the effect of different factors on the outcome variable and to control for confounding effects. Bivariate logistic regression independent variables with a *p* value < 0.25 were included in the multivariate logistic regression model. Results were reported in tabular and text form, using frequencies and summary statistics such as mean, standard deviation, and percentage to describe the study population in terms of significant variables. The strength of the association between independent and dependent variables was determined using the odds ratio with 95% CI and a crosstab.

### 2.11. Operational Definitions

#### 2.11.1. Food Safety Practices

Any aspect of the activities performed by food handlers in the preparation, transportation, storage, wrapping and packaging, and safe delivery of food to consumers.

#### 2.11.2. Food Safety Knowledge

Respondents who scored less than 70% in their responses to the questions on total food hygiene knowledge were categorized as having low knowledge. Those who scored at least 70% were considered well-informed [30, 31].

#### 2.11.3. Food Safety Attitude Levels

Respondents who scored less than 70% of their responses to all food hygiene attitude questions were rated as negative. Those who scored greater than or equal to 70% were considered have positive attitude [30, 31].

#### 2.11.4. Level of Practice in Food Safety

Respondents who obtained less than 70% of their answers on the total questions related to food hygiene practice were considered to have a low level of practice. Those who scored greater than or equal to 70% were considered to have a good level of exercise [30, 31].

## 3. Results

### 3.1. Sociodemographic Characteristics of the Respondents

A sample of 291 food handlers participated in this study with a response rate of 100%. The mean age of study participants was 29.46 years (±8.45 SD). Most (159; 54.6%) of the participants were women. Among the polyvalent pupils, 92 (31.6%) have completed primary school. However, only 155 (53.3%) of grocers received formal training in food hygiene principles in the past year. More than half (188; 64.6%) of food processors had more than one year of work experience in food processing practices. The study also points out that 157 (54.0%), 159 (54.6%), and 168 (57.7%) of the respondents, who worked more than eight hours, had poor knowledge and unfavorable attitude, respectively. Furthermore, the majority of respondents were 85 (29.2%) Orthodox Christians; eighty-nine (30.6%) declared their marital status as divorced; of the total, 86 (29.6%) were earned incomes from 2001-3000 Birr (Table 1).

### 3.2. Food Safety Practices of Food Handlers Working in the Woldia University Students' Cafeteria

This study confirmed that food processing among food processors was poor. Observational inspection and questionnaire survey indicate that 153 (52.6%) of food handlers did not wash their hands with soap and water after using the toilet; out of a total of 291 food processors, 158 (54.3%) did not respect the safety of kitchen utensils and hobs, and 162 (55.7%) of respondents showed improper storage of equipment used for food processing. In addition, categorical variables relating to food hygiene practices were indicated in Table 2.

### 3.3. Physical Hygiene Conditions of the Woldia University Students' Canteen

In the study area, the physical infrastructures such as sufficient clean water, safe storage, cold chain, sanitation, effective processing equipment, and food supply have shown unsatisfactory positions. Furthermore, 120 (41.2%) respondents answered that the number of catering spaces was two. The ventilation and lighting system of the restaurant areas was also not satisfactory. Furthermore, the categorical variables related to the physical hygiene conditions of the food areas have been listed in Table 3.

### 3.4. Main Factors Related to Food Safety Practices among Grocers Working in the Student Cafeteria of the University of Woldia

Table 4 shows the verifications of the bivariate and multivariate logistic regression analysis. The factors of not having received training in food hygiene, less than one year of work experience, poor knowledge and an unfavorable attitude, not keeping cooked food at a safe temperature, not ensuring safety of cooking utensils and surfaces, insufficient cleanliness of eating areas, not covering hair while cooking food, and failure to wash hands before beginning to handle food has been statistically associated food hygiene practices.

## 4. Discussion

The present study was designed to evaluate food safety practices and associated factors among food handlers working in Woldia University student cafeteria. The present study also provides insight into the unsatisfactory state of food processing practices in the study area. The level of good food safety practices among food handlers working in Woldia University student cafeteria was less than half (48.8%). Poor food safety practices were common in developing countries such as Ethiopia where awareness, attitudes, and lack of essential supplies do not lead to practices as expected [32]. In the study conducted in Injibara and Kessa Chewesa, Ethiopia, food processing was poor (<60%) [33] and in Gonder, Ethiopia, it was 66.4%. [32]. Moreover, the result of the present finding was much lower than other studies conducted in Indonesia (90%) [34] and Jordan (89.4%) [35]. The contradiction may be due to the context of the study area and differences in the sociodemographic characteristics of the population studied (food processors). The studies conducted in Indonesia and Jordan were conducted in healthcare settings. These facilities may have adequate equipment, and food safety procedures are stringent compared to the current study area. Furthermore, the food safety results of the present study are also lower than the studies conducted in Dessie (72%) [36], Bahir Dar 67.6% [37], and Mekelle 63.9% [38]. The differences may be due to the context of study, the environmental conditions, and the level of education of the respondents. Being a relatively hot environment in Mekelle, Bahir Dar, and Dessie can make food handlers careful when handling food. Thus, food products can be easily protected. Data from Abdi et al. [26] found that several factors such as equipment and conditions in food halls, food hygiene management, time pressure, food hygiene training, education, and food services environmental hygiene have a significant impact on food safety. The above factors are directly related to improving food safety knowledge, attitudes, and practices [29]. The level of food safety practice in the current result is higher than in the report from Nigeria 36.5% [39], the University of Gondar, Ethiopia 30.3% [40], and Gamogofa, Ethiopia 32.6% [41] and Debark 40.1% [42]. This could be due to the different study periods and thresholds used. Moreover, gender and working hour's factors show no association with food security practices. During an inspection by observation, we found that the level of hygiene of the surfaces, the frequent washing of hands, the storage of food at the right temperature, the efforts to avoid cross-contamination, the storage of equipment in places appropriate, etc. were below international standards.

Therefore, food safety aspects are crucial to ensure that desirable food properties are maintained throughout the handling, processing, and preparation phase [43]. Also, in the study area, physical infrastructure such as the adequacy of drinking water, safe storage, cold chain, sanitation, efficient treatment equipment, and catering facilities are preliminary requirements and should be standardized for safe food consumption [44]. In addition, factors related to physical infrastructure have been identified in Table 3. Physical infrastructure becomes especially important when modern food safety standards are expected to be met [45]. At the international level, the modernization of the food safety system represents an important step forward [45]. However, the improvement has come from consumers demanding safer food after high-profile disease outbreaks and contamination incidents [46]. The demand-driven basis for fundamental change is widely recognized in countries as diverse as Belgium, China, and the United States of America [45]. In the present study, it may not be an exaggeration to say that the physical environment and level of cleanliness of both the kitchen and dining room were superficial. Several factors have been reported as predictors of poor food hygiene practices among food workers (Table 4). The results of the present study showed that lack of food safety training (AOR = 2.111, 95% CI = (1.029 − 4.428)), less than or equal to one year of work experience (AOR = 3.070, 95% CI = (2.020 − 10.246)), low level of knowledge (AOR = 1.285, 95% CI = (0.125 − 0.849)), unfavorable attitude (AOR = 1.190, 95% CI = (1.361 − 9.393)), do not store cooked food at a safe temperature (AOR = 3.037, 95% CI = (1.021 − 12.096)), failure to maintain utensils and cooking surfaces (AOR = 2.022, 95% CI = (1.551 − 9.689)), inadequate cleaning of cooking areas (AOR = 2.430, 95% CI = (1.983 − 6.217)), do not cover hair while cooking food (AOR = 5.903, 95% CI = (2.243 − 9.621)), and do not wash hands before starting the food processing process (AOR = 10.019, 95% CI = (4.031 − 24.063)) showed a positive statistical association with poor food hygiene practices. The result of the present study showed that the lack of food safety training was more likely to be associated with inadequate food safety practices than people who received training. The possible justification could be that the most common form of food safety training is on-the-job training (managers have the necessary skills to adequately train their employees). Lack of incentives for managers to train their employees; training takes time and some managers may feel that training is unnecessary and so on, which affects the overall food safety training. Another reason could be the lack of training materials that can improve trainees' knowledge and behavior towards food hygiene practices. Researchers confirmed that adequate food safety training of all employees can have a positive effect on health inspection scores and some food safety behaviors such as hand washing and hair covering [47, 48]. Many food safety experts agree that worker food safety training is an essential part of preventing foodborne illness [49, 50]. Although food safety training is essential for the prevention of foodborne illnesses, it does not always lead to behavior change [51]. Additionally, food safety training helps reduce cross-contamination, improper disposal of food waste, food poisoning, and allergic reactions. Food safety training also improves employee behavior [51]. Lack of training in food hygiene is also believed to hurt practical aspects. This result is consistent with the study conducted in Addis Ababa and the city of Woldia. Out of 330 and 246 respondents, 83.8% and 85.4% in Addis Ababa and Woldia town, respectively, had not received any training in food handling and hygiene [25, 29]. Having less than or equal to one year of work experience is three times more likely to be associated with poor food hygiene practices. The possible reason could be that inexperienced grocers could not acquire better knowledge and skills related to the practice of food hygiene. More recently, Wallace et al. [52] found that work experience impacted students' perceptions of food safety knowledge and skills, but not college courses and food safety certification. On the other hand, being hotel major student had a significant impact on food safety practices, but not year of work experience [51]. This result is consistent with the study conducted in northwestern Ethiopia [53]. Similarly, a low level of knowledge shows a positive relationship with poor food safety practices compared to those with a good level of knowledge.

The possible reason could be a lack of food safety culture which may encourage the sharing of information or knowledge between managers, employees, and customers as well as the presence of attitudinal barriers in the workplace which affect practices of food safety. The transfer of food safety knowledge to employees can also be insufficient or dangerous, thus limiting the practical aspects. Grocers should be required to submit to an assessment of their knowledge in this area; and refresher courses should be provided regularly throughout employment [54]. According to Appietu's review [55], in some studies conducted elsewhere, safer food preparation and handling was best practiced by staff with at least higher education. The study in Ghana showed that the majority of street vendors had good knowledge of food safety [56]. This would help to reduce the risk of food contamination, food poisoning, and the health risk to consumers [56]. In studies conducted in many countries, better knowledge of food safety was directly linked to good food handling practices [34]. Those who are knowledgeable are expected to have a positive attitude, which is a key component of best food hygiene practices [34]. In agreement with the present study, the results reported by Abdi et al. [26], Abate et al. [29], and Dagne et al. [42] studies conducted in the cities of Addis Ababa, Woldia, and Debark found a direct link between good knowledge and better nutritional hygiene practices. Indeed, it is clear that knowledge alone is not sufficient to establish food safety practices unless other additional intervention mechanisms are used to develop positive attitudes [32]. Respondents' unfavorable attitudes were 1.2 times more likely to be associated with inferior food hygiene practices than those with a favorable attitude. The possible reason could be that attitude is the proximal factor determining translation into observable action; obviously, social predisposition hinders effective practice. The study was supported by previous surveys of public food processors in Northwestern Ethiopia (AOR = 1.97, 95% CI 1.04, 3.72) [32], and Malaysian food processors (*p* = 0.041) [32] a contradictory result was observed in an Iranian study with a significant negative correlation between attitudes and practices (rs = −0.27, *p* = 0.009) [32]. This can be attributed to scenarios where attitude change is only due to social desirability bias. Dagne et al. [42] show that those with a good attitude towards safe food handling are 3.7 times more likely to have a good level of meat handling practice. In the current study, not storing food at a safe temperature was three times more likely to be associated with unsatisfactory food safety practices than storing food at a safe temperature. Possible justification could be that grocers may not enforce guidelines to ensure proper food temperatures. Hot foods should stay hot (above 140°F) and cold foods should stay cold (below 40°F). When food is stored between 40 and 140°F (danger zone), bacteria can rapidly grow to levels that can cause illness. A similar study showed that a favorable temperature promotes the rapid growth of bacteria, increasing their numbers to the point where some can cause disease [57]. Failure to follow utensil and cooktop safety was also 2 times more likely to be associated with inadequate food safety practices than maintaining utensil and cooktop safety. This may be because if worktops and utensils are not kept clean, bacteria can spread into food and make consumers ill. Therefore, always wash both utensils and countertops thoroughly after touching raw foods.

Similarly, the study by Idris et al. [58] reported that utensils should be disinfected and stored in a clean and protected place and that proper handling should be achieved to the highest standard. The study also found that dirty cooking surfaces are a possible cause of chemical and microbiological food poisoning. In the present study, insufficient cleaning of food service areas was 2 times more associated with unsanitary food handling practices than adequate cleaning of food service areas. The reason could be insufficient general cleanliness of the kitchen, which potentiates food adulteration. Poor food hygiene practices and unsanitary locations of eating rooms and equipment were the leading causes of foodborne infections and deaths [57, 58]. In order to minimize foodborne illnesses, shareholders should adopt good food handling and safety practices. Additionally, in the present study, do not cover hair while cooking food was 5 times more likely to be associated with poor food handling practices than covering hair. The possible explanation could be that hair is not tied up or uncovered, it is more likely to fall into food, and employees are more likely to touch their hair. This allows bacteria to be transferred to food, especially if it is unpackaged. Human hair is both a physical and microbiological contaminant as it can lead to the growth of microorganisms in food along with the foreign substance. These contaminants enter the hair from the environment and therefore the same toxic substances from human hair can enter the food. Finally, not washing hands before starting food handling was the last but not least factor associated with unhygienic food handling practices 10 times more often than washing hands before starting food handling food. Possible reasons for this could include time constraints, inadequate facilities, lack of accountability, and a general lack of support for hand washing practices in the workplace, which has hampered food safety practices. Harmful bacteria such as *E. coli*, *Salmonella*, *Staphylococcus aureus,* and viruses (e.g., norovirus) present on the hands of food handlers can contaminate food unless removed with proper hand washing techniques [57, 59]. Therefore, hand washing is essential to prevent food handlers from contaminating food. Despite contributions to current knowledge on food hygiene practices, this study also has some limitations, such as being localized (Woldia University) and a small sample size could limit the reliability and generalizability of the results. The cross-sectional nature of the study design also limits the applicability of the findings in establishing causality between variables.

## 5. Conclusions

In summary, more than half (51.2%) of respondents (food processors) may have used poor food safety practices. Various factors including no training in food hygiene, less than one year of professional experience, little knowledge and unfavorable attitude, not keeping cooked foods at the right temperature, not paying attention to the safety of utensils and cooking surfaces, cleanliness insufficient food service facilities, not covering hair when cooking food, and not washing hands before beginning to handle food are significantly associated with poor food safety practices. In contrast, gender and work hours show no association with food hygiene practices. Finally, relevant agencies or stakeholders can use this finding to address gaps identified through health education programs and environmental health services, such as periodic inspections, effective enforcement of food safety laws and regulations, and increased capacity of food processors through training. In addition, a HACCP (Hazard Analysis Critical Control Point) framework is required for the purpose of analyzing and controlling biological, chemical, and physical hazards from the production, procurement, and processing of raw materials to the production, distribution, and consumption of the final product. Finally, future studies should focus on counting bacteria and protozoa from unsanitary foods and utensils.

## Figures and Tables

**Table 1:** Sociodemographic characteristics of food workers working at the Woldia University canteen, May-July 2021, (*n* = 291).

Variables	Food operators response		
	Categories	Frequency	Percentage
Gender	Male	132	45.4
	Female	159	54.6

Age	≤29	115	39.5
	≥30	176	60.5

Level of education	No formal education	58	19.9
	Primary education	92	31.6
	Secondary education	74	25.4
	Higher education	67	23.0

Receive food safety training	Yes	136	46.7
	No	155	53.3

Religion	Orthodox Christianity	85	29.2
	Muslim	83	28.5
	Protestant	68	23.4
	Specify others	55	18.9

Working experience	≤1 year	103	35.4
	>1 year	188	64.6

Marital status	Married	86	29.6
	Single	60	20.6
	Divorced	89	30.6
	Others say	56	19.2

Income^∗^	≤1000	64	22.0
	1001-2000	70	24.1
	2001-3000	86	29.6
	≥3000	71	24.4

Working time	≤8	134	46.0
	>8	157	54.0

Knowledge	Good	132	45.4
	Poor	159	54.6

Attitude	Favorable	123	42.3
	Unfavorable	168	57.7

^∗^ = Birr (in Ethiopia). Source: survey data result, 2021.

**Table 2:** Grocer responses regarding food safety practices in the Woldia University students' canteen, May-July 2021 (*n* = 291).

Variables	Categories	Grocer responses	
		Frequency	Percentage
Do you wash your hands with soap and water after going to the toilet?	Yes	138	47.4
	No	153	52.6

Do you keep cooking utensils and cooking surfaces safe?	Yes	133	45.7
	No	158	54.3

How do you rate the storage of equipment used for food processing?	Correctly placed	129	44.3
	Improper storage	162	55.7

Do you cut your nails regularly?	Yes	154	52.9
	No	137	47.1

Do you cover your hair while cooking?	Yes	150	51.5
	No	141	48.5

Do you wash your hands before you start handling food?	Yes	156	53.6
	No	135	46.4

Do you wash utensils with three or more compartments?	Yes	142	48.8
	No	149	51.2

Do you wash dishes with soap or detergent?	Yes	177	60.8
	No	114	39.2

Keep cooked foods at a safe temperature?	Yes	127	43.6
	No	164	56.4

Source: survey data result, 2021.

**Table 3:** Physical sanitary conditions of food premises at Woldia University students' canteen, May–July 2021 (*n* = 291).

Variables	Categories	Food handlers response	
		Frequency	Percentage
Number of dining rooms	One room	103	35.4
	Two-piece rooms	120	41.2
	≥Three rooms	68	23.4

Number of windows in dining room	One window	114	39.2
	Two windows	99	34.0
	≥Three windows	78	26.8

Floor of gastronomy rooms	Soil	119	40.9
	Cement	172	59.1

Ventilation system for dining rooms	Satisfactory	127	43.6
	Unsatisfactory	164	56.4

Light system for dining rooms	Satisfactory	124	42.6
	Unsatisfactory	167	57.4

Cleanliness of dining rooms	Sufficient	107	36.8
	Insufficient	184	63.2

Water consumption at grocers	Private line	125	43.0
	Common pipe	166	57.0

Water-related conditions	Latrine water segregation	114	39.2
	Has no water separation	177	60.8

Cleanness of latrine	Clean	138	47.4
	Impure	153	52.6

Liquid waste disposal system	Open field	186	63.9
	Use segregation	105	36.1

Solid waste disposal methods	Garbage dump	154	52.9
	Open field	137	47.1

Source: survey data result, 2021.

**Table 4:** Bivariate and multivariate analysis of factors related to food safety practices among food workers working in the student canteen at the University of Woldia, May-July 2021, (*n* = 291).

Variables	Status of food safety practices		OR (95% CI)		
	Good (%)	Poor (%)	COR (95% CI)	*p* value	AOR (95% CI)
Gender					
Male	22(7.6)	110(37.8)	0.065(0.036-0.116)	.000	0.457(0.142-1.471)
Female	120(41.2)	39(13.4)	1.00		1.00
Food safety training received					
No	31(10.7)	124(42.6)	1.056(1.031-8.101)	.000	2.111(1.029-4.428)^∗^
Yes	111(38.1)	25(8.6)	1.00		1.00
Working experience					
≤1 year	58(19.9)	45(15.5)	1.596(1.983-5.589)	.058	3.070 (2.020-10.246)^∗∗^
> 1 year	84(28.9)	104(35.7)	1.00		1.00
Working time					
≤8 hours	27(9.3)	107(36.8)	0.092(0.053-0.160)	.000	0.942(0.330-2.683)
> 8 hours	115(39.5)	42(14.4)	1.00		1.00
Level of knowledge					
Poor	48(16.5)	111(38.1)	3.175(2.105-7.910)	.000	1.285(0.125-0.849)^∗^
Good	94(32.3)	38(13.1)	1.00		1.00
Level of attitude					
Unfavorable	56(19.2)	112(38.5)	2.150(1.130-3.155)	.000	1.190(1.361-9.393)^∗∗^
Favorable	86(29.6)	37(12.7)	1.00		1.00
Keep cooked food at a safe temperature?					
Yes	68(23.4)	59(20.3)	1.00		1.00
No	74(25.4)	90(30.9)	0.432(0.035-0.641)	.213	3.037(1.021-12.096)^∗∗^
Do you keep cooking utensils and cooking surfaces safe?					
Yes	65(22.3)	68(23.4)	1.00		1.00
No	77(26.5)	81(27.8)	1.031(1.062-7.807)	.021	2.022(1.551-9.689)^∗^
Cleaning of cooking areas					
Sufficient	55(18.9)	52(17.9)	1.00		1.00
Insufficient	87(29.9)	97(33.3)	0.682(0.715-0.947)	.130	2.430(1.983-6.217)^∗^
Do you cover your hair while cooking?					
Yes	86(29.6)	64(22.0)	1.00		1.00
No	56(19.2)	85(29.2)	0.025(0.002-0.078)	.000	5.903(2.243-9.621)^∗^
Do you wash your hands before handling food?					
Yes	80(27.5)	76(26.1)	1.00		1.00
No	62(21.3)	73(25.1)	2.046(1.054-9.332)	.003	10.019(4.031-24.063)^∗∗^

Statistically significant at ^∗^ = *p* < 0.05; ^∗∗^ = *p* < 0.001; 1.00 = reference groups; COR: crude odds ratio; AOR: adjusted odds ratio; CI: confidence interval; Source: survey data results, 2021.

## Data Availability

The data used to support the findings of this study are included within in the article.

## Supplementary Materials

Supplementary Materials All supporting information has been provided as follows: Table S1: sociodemographic characteristics of study subjects; Table S2: food safety practices of food operators; Table S3: physical sanitary conditions of the food premises; S4: questions used to measure respondents' food safety level of knowledge; S5: questions used to measure respondents' food safety level of attitude; S6: focus group and interview check lists; S7: observation checklist.
