# Supplementary material for: Food Safety Practices and Associated Factors in Food Operators: A Cross-Sectional Survey in the Students' Cafeteria of Woldia University, North Eastern Ethiopia
Source: Int J Food Sci. 2022 Dec 9;2022:7400089. doi: 10.1155/2022/7400089 (PMC9757932; doi:10.1155/2022/7400089)
Supplement: Supplementary Materials — All supporting information has been provided as follows: Table S1: sociodemographic characteristics of study subjects; Table S2: food safety practices of food operators; Table S3: physical sanitary conditions of the food premises; S4: questions used to measure respondents' food safety level of knowledge; S5: questions used to measure respondents' food safety level of attitude; S6: focus group and interview check lists; S7: observation checklist. [file 7400089.f1.docx]

**Supplemental Material 1**

**Questionnaires used for participants’ data collection**

**WOLDIA UNIVERSITY
Faculty of Natural and Computational Science
Department of Biology**

The purpose of this questionnaire is to gather information/data for research conducted under the title of “**FOOD SAFETY PRACTICES AND ASSOCIATED FACTORS IN FOOD OPERATORS: A CROSS- SECTIONAL SURVEY IN THE STUDENTS’ CAFETERIA** **OF WOLDIA UNIVERSITY, NORTH EASTERN ETHIOPIA.”** This questionnaire consists of questions that focus on the socio-demographic characteristics of the respondents, food safety practices of food handlers, physical sanitary conditions of the food premises at Woldia University students’ cafeteria, knowledge and attitude level of the respondents. The success of this study depends on the quality and trustworthiness of the responses given by the research participants (respondents). You are kindly requested to answer the questions carefully for the successful completion of this research. Finally, I would like to express my appreciation for your willingness to be part of this research work.

Thank you!

**PART-I: QUESTIONS RELATED TO SOCIO-DEMOGRAPHIC CHARACTERISTICS**

**Give your answer for the following questions by using a tick mark “**✓**“in the box of your choice**

| **No** | **Questions** | **Response** |
| --- | --- | --- |
| **1** | Age | ___________ (in years) |
| **2** | Gender | 1. Male   2. Female |
| **3** | Level of educational | 1. No formal education 2. Primary education 3. Secondary education 4. Higher education |
| **4** | Received food safety training | 1. Yes  2. No |
| **5** | Religion | 1. Orthodox Christianity 2. Muslim 3. Protestant   4. Specify others………………. |
| **6** | Working experience | 1.≤ one year  2. > one year |
| **7** | Marital Status | 1. Married  2. Never married  3. divorced  4. Others (specify)……………………. |
|  |  |  |
| **8** | Income | 1. ≤1000 2. 1001- 2000 3. 2001- 3000   4. ≥3000 |
| **9** | Working time | 1. ≤ 8  2. >8 |

**PART-II: Food safety practices of food operators working in Woldia University students’ cafeteria**

| **No** | **Question** | **Response** |
| --- | --- | --- |
| **1** | Do you wash your hands with soap and water after going to the toilet? | 1. Yes  2. No |
| **2** | Do you keep cooking utensils and cooking surfaces safe? | 1. Yes  2. No |
| **3** | How do you rate the storage of equipment used for food processing? | 1. Correctly placed  2. Improper storage |
| **4** | Do you cut your nails regularly? | 1. Yes  2. No |
| **5** | Do you cover your hairs while cooking? | 1. Yes  2. No |
| **6** | Do you wash your hands before you start handling food? | 1. Yes  2. No |
| **7** | Do you wash utensils with three or more compartments? | 1. Yes  2. No |
| **8** | Do you wash dishes with soap or detergent? | 1. Yes  2. No |
| **9** | Keep cooked foods at a safe temperature? | 1. Yes  2. No |
| **PART-III Physical sanitary conditions of the food premises at Woldia University students’ cafeteria** | | |
| **1** | Number of dining rooms | 1. One room  2. Two-piece rooms  3. Three and above |
| **2** | Number of windows in dining room | 1. One window  2. Two windows  3. Three and above windows |
| **3** | Floor of gastronomy rooms | 1. Soil  2. Cement |
| **4** | Ventilation system for dining rooms | 1. Satisfactory  3. Unsatisfactory |
| **5** | Light system for dining rooms | 1. Satisfactory  2. Unsatisfactory |
| **6** | Cleanliness of dining rooms | 1. Sufficient  2. Insufficient |
| **7** | Water consumption at grocers | 1. Private line  2. Common pipe |
| **8** | Water related conditions | 1. Latrine water segregation  2. No water separation |
| **9** | Cleanness of latrine | 1. Clean  2. Impure |
| **10** | Liquid waste disposal system | 1. Open field  2. Use segregation |
| **11** | Solid waste disposal methods | 1. Garbage dump    2. Open field |

**Part IV: Questions used to measure respondents’ food safety level of knowledge.**

1. Did you know about food hygiene?

Yes No

2. Did you know about personal hygiene?

Yes No

3. Did you know foods can be contaminated by disease causing pathogens?

Yes No

4. Did you know about food borne diseases?

Yes No

5. Did you know about safe food handling?

Yes No

6. Did you know about person to person food borne disease transmission?

Yes No

7. Did you know about hand washing procedures?

Yes No

8. Did you know about food poisoning?

Yes No

**Part V: Questions used to measure respondents’ food safety level of attitude.**

1. Hand washing is imperative to keep foods safe

Agree Disagree

2. Personal hygiene is the preliminary action during food handling processes

Agree Disagree

3. Reducing cross contamination between groups or individuals increases food safety

practices. Agree Disagree

4. Keeping cooked foods in safe place and temperature saves food from contamination by

bacteria. Agree Disagree

5. Sanitizing catering rooms and food contact surfaces are recommended for food hygiene practices.

Agree Disagree

6. Using safe water is mandatory while cooking foods in catering rooms

Agree Disagree

7. Cleanness and distance of latrine from catering is a decisive factor for food safety

Agree Disagree

8. Wastes should properly disposed in the right places

Agree Disagree

**Part Six: Focus group and interview check lists**

***Opening questions***

How long have you worked in Woldia University? What are your general thoughts on working here?

***Questions to be raised during the focus group discussion and interview***

1. Do you think that food safety is necessary for consumers’ health?

2. Do you think that a number of factors that can affect food safety practices? Explain

3. Does your manager encourage/discourage food safety practices? Explain why

4. In your opinion are there any physical constraints that affect food safety practices in your work place?

5. In your opinion are there any risks that my contaminate food and drink establishments in your working area?

6. What does “food safety practices” mean to you? If I say the word food safety, what do you think about it? What are your opinions about taking care of food adulteration? Do you believe food adulteration can be dangerous for life and if so, in what way and to whom?

7. Do you look differently on different aspects of food contamination and in what way? Do you know food safety practice? Does it a cautious step or not?

8. Are you aware of some health effects that can be caused by contaminated food, e.g., diarrhea, dehydration and even death?

9. What are your personal thoughts on food safety? Do you think that it is important to encourage food safety practices in wider perspective (larger community)?

**Part seven- Observation checklist**

1. Does the manager undertake periodic food safety inspection?

| 2. Do the stakeholders involved in food safety practice? |
| --- |

3. Do the physical sanitary conditions and cafeterias kept well?

4. Does the university have a good waste disposal site?

5. Do the catering rooms have a good ventilation rooms?

6. Are there any cautious practices by the food handlers to safeguard food safety?

7. Does the storage temperature range is in agreement with the international standard.

8. Do all shareholders responsible for food safety form distribution to table consumption?

9. Do the food handlers trimming their finger, frequently washing their hands and covering their hair while preparing foods?

**Source:** Adapted from Abdi et al [26] and Abate et al [29]
